# Evaluating the enhancement and improvement of China’s technology and financial services platform innovation strategy

**DOI:** 10.1186/s40064-016-3562-x

**Published:** 2016-11-03

**Authors:** Ching-Sung Wu, Kuang-Hua Hu, Fu-Hsiang Chen

**Affiliations:** 1 College of Business, Chinese Culture University, No. 55, Hwa Kang Rd., Yang Ming Shan, Taipei, 11114 Taiwan; 2 Accounting School, Nanfang College of Sun Yat-sen University, Wenquan Town, Conghua, Guangzhou, 510970 China; 3 Department and Graduate School of Accounting, Chinese Culture University, No. 55, Hwa Kang Rd., Yang Ming Shan, Taipei, 11114 Taiwan

**Keywords:** Innovation, Technology and finance, Investment and finance, Credit rating, Credit guarantee, Multiple attribute decision making (MADM)

## Abstract

The development of high-tech industry has been prosperous around the world in past decades, while technology and finance have already become the most significant issues in the information era. While high-tech firms are a major force behind a country’s economic development, it requires a lot of money for the development process, as well as the financing difficulties for its potential problems, thus, how to evaluate and establish appropriate technology and financial services platforms innovation strategy has become one of the most critical and difficult issues. Moreover, how the chosen intertwined financial environment can be optimized in order that high-tech firms financing problems can be decided has seldom been addressed. Thus, this research aims to establish a technology and financial services platform innovation strategy improvement model, as based on the hybrid MADM model, which addresses the main causal factors and amended priorities in order to strengthen ongoing planning. A DEMATEL technique, as based on Analytic Network Process, as well as modified VIKOR, will be proposed for selecting and re-configuring the aspired technology and financial services platform. An empirical study, as based on China’s technology and financial services platform innovation strategy, will be provided for verifying the effectiveness of this proposed methodology. Based on expert interviews, technology and financial services platforms innovation strategy improvement should be made in the following order: credit guarantee platform (*C*)_credit rating platform (*B*)_investment and finance platform (*A*).

## Background

Since the implementation of open policy following its reform in 1978, China’s remarkable economic achievement has attracted world attention, and has continued to develop rapidly. In 2010, China became the world’s second largest economy. However, though the envy of the world, China’s speed of economic development and wealth accumulation have also exacted a heavy price. Its economic development is locked in a high energy consumption and low efficiency mode, and its persistent low status in the global industrial chain, excessive resource consumption and severe environmental pollution have become intolerable (Yan and Huang 2013).

In the mean time, the world’s major developed countries have increased their investment in technological innovation, focused on basic research, and actively developed new energy, new materials, environmental protection and other emerging industries, and have seized the pinnacle of international economy and technology. Evidently, technological innovation is critical in global competition (Wang and Xu 2012). Therefore economic development must transform and adjust its mode by promoting technological innovation and entrepreneurship to enhance the international competitiveness of enterprises and industries. Technological systems and management must be reformed and become innovative, and financial innovation must be introduced and encouraged. As such, the integration of technology and finance is a key intervention point for unifying technology and economy (You and Zhu 2011). With accelerating technological innovation in China, the role of technology and finance is becoming more prominent. In recent years, the government of China has formulated pluralistic technology and finance policies involving many departments such as the Ministry of Science, the People’s Bank of China, the Ministry of Finance, the China Banking Regulatory Commission, and the Securities Regulatory Commission to promote technological innovation, define the role of technology and finance development in providing financial services to technological innovations, and resolve funding needs for transforming and industrializing technological innovations (Wang and Xu 2012).

Technology and finance is the in-depth integration of technology and financial innovation activities. It is an overall term for activities that promote technological innovation and the development of technological industry where in order to integrate technological innovation and financial capital, governments, financial institutions, investors and other financial resources providers offer various capital, innovative financial products, financial policy and financial service to enterprises or institutions that develop, transform outcomes and industrialize technology innovation (Xu et al. 2011). Establishing an intermediary service system for technology and finance is an important part of integrating science and finance. The Chinese government has already established an initial technology and financial service platform. For example, with “surplus power” as its core, the Chengdu High-tech Zone has created an investment and finance platform for Small and Medium Enterprises (SMEs), and the Suzhou Industrial Park has established the first national loan platform for technological SMEs. In addition, areas such as the Zhongguancun Science Park, Pudong New District, Wuxi High-tech Zone have also set up technology and finance service groups, technology venture capital groups and technology and finance service centers. These platforms are becoming effective technology and financial service platforms for connecting technological and financial resources, and provide highly effective and comprehensive investment and finance service for technology industry by attracting all kinds of financial and intermediary institutions (Ma 2014).

Technology and financial service platforms mainly provide investment and finance service, technology innovation guidance, and comprehensive and integrated financial service (You and Zhu 2011). In terms of investment and finance, relevant Chinese government departments (usually the technology sector, investment sector and financial sector) explore the technical standards, profitability, and market prospects and potential of technological industries according to development policies for promoting technological innovation industries. Promising technology industries and technological innovations are recommended to investment and financial institutions. Through these technology and financial service platforms, investment and financial institutions provide financial resources for innovation according to the needs of the technology companies. In terms of technology innovation guidance, the Risk Investment Funds, SME Guarantee Agency and Petty Loan Institutions of technology and financial service platforms give priority to high-tech new technology enterprises, and high technology growth enterprises are supported through “equity”, “loans” and “guarantee”. In particular, policy high-tech industries are given funding during their “start-up” and “development period” to maximize the effect of financial capital on technological expansion (Perez 2002). In terms of providing comprehensive integrated finance services, technology and financial service platforms converges the comprehensive and integrated financial services that banks, securities, insurance and trust funds and other financial institutions provide for enterprises, which also enhances cooperation among the financial institutions and unify development.

The local Chinese governments exert their organizational coordination and supervisory capacity to establish regional technology and financial service platforms. Through these platforms, new high-tech enterprises can quickly grasp the regulations and policies of various government levels, submit their development needs and advantage, and communicate effectively with financial institutions to obtain financial resources for expanding their operations. Financial institutions can quickly discern the credit information and financial needs of high-tech and new technology industries, and at the same time integrate the results of external credit ratings to more effectively control credit risk and reduce credit cost, thereby generating technology innovation profit on the financial capital (Perez 2002). Through technology and financial service platforms, the government can also learn more about the development needs of high technology and new technology enterprises, and further revise relevant government policies and regulations, such as standardizing guarantee markets and developing credit rating markets to provide a better financial environment. In summary, China’s regional technology and financial service platforms innovation strategy can primarily be categorized into “Investment and Financial Platform”, Credit Rating Platform” and “Credit Guarantee Platform” (Ma 2014; Wang and Xu 2012; Xu et al. 2011; You and Zhu 2011).

While high-tech firms in developing countries play a significant role in job creation and national economic development, it is difficult to obtain financing for them (Xiong et al. 2010; Huda 2012). In order to assist the growth of high-tech firms, the European Union set up a special financing institution for high-tech firms, including the European investment bank and the European investment fund. The European investment bank provided concessional loans for entrepreneurial firms or high-tech firms in the expansion phase; while the European investment fund was the first international high-tech firms’ credit guarantee institution, which afforded a guarantee to small businesses, with employees not exceeding 50, in information and communications technology investment. The China Banking Regulatory Commission (CBRC 2015) also founded the China Association of Microfinance Companies on January 30, 2015, and one of its main works is to promote the exploration of techniques and product innovation related to high-tech firms loans, in order to lower the funding costs of high-tech SMEs, improve loan quality and efficiency, and increase the innovative development of the microfinance industry.

Although the design of a comprehensive technology and financial services platform is very important, it is not an easy task. Prior studies of technology and financial service platforms primarily put greater focus on the construction and operation of technology and financial service platforms (You and Zhu 2011; Ma 2014); the synergy of technology innovation and technology and finance (Wang and Xu 2012), and the coupling coordinated degree evaluation of regional sci-tech innovation and technology and finance (Xu et al. 2011). Fewer studies addressed how microfinance for high-tech enterprises should be designed to fit any finance market, and how high-tech enterprises development should be designed by considering factors related to lending channels and credit risks. Reviewing the above technology and financial services platform studies shows that the focus was on unilateral considerations, and while those factors did not exhaustively improve the credit environment, they are based on the assumption of conventional statistical techniques (Park and Ren 2001; Xiong et al. 2010; Abroud et al. 2013), with some restrictions, such as linearity, normality, and independence. Given that such assumptions are often inconsistent with the means for improving technology and financial services platform factors, these methods have their intrinsic limitations in terms of effectiveness and validity. In fact, technology and financial services platform factors are not independent, and among them there exists an influential interrelationship (dependence and feedback); meanwhile, Multiple Attribute Decision Making (MADM) approaches can be applied to solve this problem (Lu et al. 2015) While this method has been widely applied in various fields, it was seldom used by financial industries, such Hu et al. (2015), Shen et al. (2014) and Wu et al. (2010). Therefore, in order to improve the technology and financial services platform, this paper considered all related variables (called dimensions/criteria) of microloans and risk precaution, as based on Guangdong’s development of technology and financial services as the empirical case.

Thus, this paper proposes a novel hybrid MADM framework for Guangdong’s technology and financial services platform, which combines the Decision Making Trial and Evaluation Laboratory (DEMATEL) technique to determine a total influence matrix of dimensions and criteria, and construct an influential network relationship map (INRM) to identify the influential levels of different dimensions/criteria. The concept of Analytic Network Process (ANP) and the total influence matrix of DEMATEL would be applied to derive the influencing weights of DANP (DEMATEL-based ANP). Then, the performance of dimensions and criteria will be evaluated using the modified VIKOR and DANP. After the influential factors/criteria are introduced, the aspired technology and financial services platform of Guangdong will be surveyed again to ensure improvement.

This novel hybrid MADM proposed for solving real-world technology and financial services platform problems improves upon the previous methods in the following ways: (1) providing interdependency model according to DANP concepts to overcome problems of dependence and feedback among technology and financial services platform factors; (2) incorporating modified VIKOR approaches to prioritize improvements technology and financial services platform factors according to the aspiration levels, allowing the proposed method to avoid selecting the seemingly best solution from among inferior choices, options or alternatives. Instead, all factors were replaced by aspiration levels obtained from modified VIKOR; (3) offering strategy improvement planning models that give decision-makers multiple solutions for technology and financial services platform factors improvement priorities. Hence, the novel hybrid MADM shifts the focus from simple ranking and selection of the most preferable factors or alternatives to performance improvements and methods.

The remainder of this paper is organized, as follows. In section “Literature review”, the assessed dimensions and criteria are introduced. In section “Building a novel MADM model for technology and financial services platform improvement”, a MADM based analytic framework and methods by introducing the DEMATEL technique and modified VIKOR are proposed for constructing the technology and financial services platform. Section “An empirical case: an improvement plan for Guangdong technology and financial services platform” presents an empirical study of Guangdong’s finance services selection of high-tech enterprises, and its aspired level of technology and financial services achievements will be provided. Section “Conclusions and remarks” offers conclusions with observations and remarks.

## Literature review

The development of technology and finance is often associated with financial risk. High-tech enterprises belong to an unstable, immature, and fast changing industry, in comparison with others. Whether it is the demands of technology financing or the supply side, intermediaries and governments are faced with different risks. For long-term stable development of technology and financial services, the demands of high-tech enterprise investment and financing, as well as the related issues of corporate credit risk, must be prudently considered. The investment and financial platform, credit rating platform, and credit guarantee platform, are all crucial components of technology and financial services.

### Investment and financial platform

In the start-up and expansion stages, high-tech enterprises require the support of high-tech loans and venture capital; therefore, high-tech microfinance institutions are an important pillar for the development of science and finance. Relative to large firms, start-up stage high-tech businesses do not have an abundance of assets that can be evaluated and pledged as collateral, and tend to have few transaction records, little credit history, or information transparency (Berger and Udell 1998). In view of this situation, most high-tech enterprises received their external funding through private equity and the debt market, rather than public markets (Berger and Udell 1998; Kaivanto and Stoneman 2007). While African microenterprise credit programs have increased in the last decade, there appears to be little evidence of increased income or levels of employment, thus, it is undeniable that in some cases microcredit can stimulate enterprise growth and improve performance (Buckley 1997). For high-tech enterprises and new public firms in developing countries, public equity finance is a useful and appropriate loan approach (Pretes 2002; Brown and Petersen 2010). Bose (2005) also argued a positive relationship between equity finance and economic growth. Bystrӧm (2008) believed that credit derivatives (such as collateralized debt obligations) could extend the finance market to microenterprises/high-tech enterprises, and accelerate loans growth. Cumming (2007) proposed that the Australian governmental innovation investment funds foster the development of start-up and high-tech firms. Due to advances in information technology and the development of financial markets, the high-tech enterprise financing network is a trend based on professional personnel, which could provide finance related information, enhance the efficiency of financing, and promote the high-tech industry.

### Credit rating platform

The databases of banks have accumulated large quantities of information regarding the finances, payment records, and creditworthiness of clients. Banks can conduct a credit risk assessment, endow different credit terms for individuals, and conduct credit crisis prevention dependent on such databases (Oreski et al. 2012; Agier and Szafarz 2013; Bartoli et al. 2013; García et al. 2013). Huda (2012) indicated that the government should construct databases of high-tech firms in order to reduce asymmetric information, and act as an agency for banks in their financial distributions to high-tech firms that lack financing. Avery et al. (2004) showed that credit databases can be used to build a consumer credit model, which should incorporate situational data (such as temporary economic and personal shocks), in order to enhance the potential effectiveness of such models. Voordeckers and Steijvers (2006) used a database of SMEs (high-tech firms are included in SMEs) credit approval rates from a Belgian bank to show that stronger creditor protection regarding business collateral and personal commitments would be conducive to the approval of credit and better loan terms (Berger and Udell 1995). Zhu and Zhang (2012) also pointed out that, data preprocessing of customer credit cards can improve the prediction accuracy of risk detection.

A credit rating database offers assessments regarding borrowers’ creditworthiness, investment risk, and default probability for financial institutions (Chen and Cheng 2013). Akdemir and Karsli (2012) suggested that, while credit rating agencies play a praiseworthy role in Eurozone financial markets, if the reputation of an agency was suspect, and did not provide correct ratings, it may elevate global financial market crisis. Credit rating agencies provide rating reviews that are influenced by the informational content of crediting ratings, which possibly extend their economic function in financial markets (Bannier and Hirsch 2010). Due to higher uncertainty and lower institutional quality in emerging markets, rating effects have stronger effects (Erbas 2005). A regulator could provide an incentive to deviate from credit rating agencies and offer inflated ratings, thus, an approval scheme exists to lead to correct ratings (Stolper 2009). There exists a financial system with a perfect credit rating that microfinance institutions were more than willing to expand the frontier of finance by providing loans to high-tech enterprises (Hartarska and Nadolnyak 2008). Property appraisal estimates from external or internal appraisers were different due to information asymmetry; however, external appraiser agencies are better able to provide a reasonable assessment of the fair value of assets (Muller and Riedl 2002).

### Credit guarantee platform

While most countries seek to encourage high-tech firms’ growth, essentially expansion capital able to support substantive growth have less access to debt financing, as compare with larger firms (Riding et al. 2001; Beck et al. 2005). However, Crowling (2010) argued that banks would not lend to high-tech enterprises in the first instance in the absence of a loan guarantee, thus, the available finance of small firms was constrained to an imperfect capital market. Riding et al. (2001) found that loan guarantee programs is an effective means of supporting start-up and risky enterprise expansion in Canada, and similar schemes are in effect in Japan, the U.K., Korea, and Germany, which can create a large number of jobs. Columba et al. (2010) proposed that, while it is difficult for high-tech enterprises with a short repayment record and credit history to obtain loans from banks, their borrowing capacity may be improved by joining mutual guarantee institutions (MGIs) that have better screening and monitoring ability in borrowers. At the peak of crisis, if small firms with joined MGIs did not appear financial tensions, however, the information from MGIs which can be rating systems (Bartoli et al. 2013). A well-designed government-sponsored credit guarantee scheme should consist of credit rating databases, limit the ratio of guaranteed loans to total loans, and has an affordable ceiling on total budget (Honohan 2010). According to Beck et al. (2010), the largest loan guarantee amount in Asia is approximately $ 41.1 billion, which is almost 5% of the GDP, where 45% of these funds were established to assist SMEs and 5% to new business.

In this paper, there are advantages and disadvantages of selection model/technological model as followers. First, in terms of investment and finance platform, (1) Advantages: The found of investment and finance platform is a construction of sound databases and multiple channel choice in financing services. It provides a diversified investment and financing channels for high-tech firms. The government, financial institutions and high-tech enterprises have a smooth communication, and enterprises are capable quickly to get government supports and financing helps and solve their financing problems. It could greatly save the cost of financing for business, and can enhance the financing efficiency of regional technology and financial services platform. (2) Disadvantages: Even if there are diversified pipeline of investment and financing platform for high-tech companies, it is detrimental to the establishment of technology and financial services platform if it is not proper to control risk and post-loan monitoring.

Second, in terms of credit rating platform, (1) Advantages: The fundamental reason of financing difficult problem for high-tech enterprises is information asymmetry. For the banks, lending to high-tech companies, accessing their information, post-loan supervision costs and bearing market risks are higher than loan to large enterprises. In this case, if there is no effective mechanism to help banks to measure credit risk of high-tech firms and reduce the cost of access to enterprise information, even if the bank with sufficient funds is not willing to take risks in loans. So it is an effective mechanism to establish a credit rating platform of high-tech firms. It is conducive to investors to reduce or avoid investment risks due to the information asymmetry through the use of credit scoring to evaluate high-tech business loan applications. And the credit scoring is in favor of financial institutions to make credit decisions for high-tech enterprises, and take appropriate risk prevention measures and help the construction of technology and financial services platform. (2) Disadvantage: If the credit rating agencies could not provide a fair, transparent and correct credit rating, it would be exacerbated information asymmetry. There will affect the borrowers and the lenders’ willingness to investment and financing, then a complete and sustainable technology and financial services platform would not be constructed.

Third, in terms of credit guarantee platform, (1) Advantages: Because of the characteristics of smaller capital and unstable operating for high-tech enterprises it leads to the difficulty to obtain bank credit support. The development of various types of credit guarantee institutions has become an important means to solve the financing difficulty. Therefore, construction of credit guarantee platforms can become a milestone in fostering sustainable development of high-tech enterprises. (2) Disadvantages: In general, high-tech guarantee institutions in providing loan guarantee for high-tech companies, they will be required their own patent collateral to pledge to guarantee agencies. But how to correctly assess the value of the intellectual property rights remains a difficult and complex technique, and also increasing the difficulty of establishment of high-tech credit guarantee platform.

According to above mention, investment and finance platform represents the first dimension, which explores how the technology and financial services platform is established (Kaivanto and Stoneman 2007). The second dimension focuses on credit rating platform, indicating how credit databases can be used to create a credit rating system (García et al. 2013). The last dimension, credit guarantee platform, refers to whether guarantee institution have been properly protected to provide services (Riding et al. 2001). Several studies (Perez 2002; You and Zhu 2011; Wang and Xu 2012; Ma 2014) indicate that technology and financial services platform is influenced by three dimensions: investment and finance platform, credit rating platform and credit guarantee platform.

## Building a novel MADM model for technology and financial services platform improvement

**Fig. 1:**
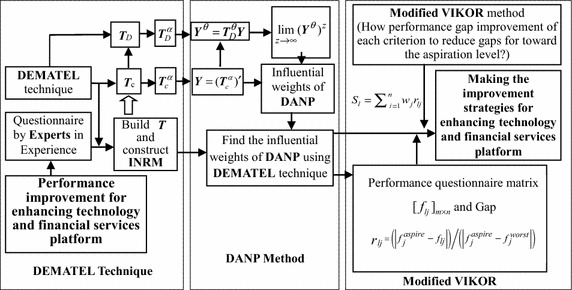
Model procedure using new hybrid MADM for making the technology and financial services platform

The MADM model is launched based on the above-mentioned studies, and is considered an appropriate method for the evaluation of technology and financial services platform; the outcome for improvement performance in each criterion can be referenced by decision-makers. This section consists of four sub-sections. First, the data collection process is introduced. Secondly, the DEMATEL method is demonstrated, and how to construct an INRM is proposed. Thirdly, this study illustrates how to derive the influential weights of DANP, as based on the total influence matrix of DEMATEL. Finally, the modified VIKOR method is applied to transform the performance values into the amended gap. An analytical framework for technology and financial services platform can be illustrated as Fig. 1.

### Data description

This paper, professional personnel, and scholars with a wealth of knowledge regarding technology and financial services will be considered for providing research samples through personal interviews and questionnaire surveys. The viewpoints of respondents were collected, the effects of the assessment criteria of the three dimensions are depicted regarding the technology and financial services platform; 10 pre-test questionnaires were assigned, including 3 government officials from the Guangdong office, the China Banking Regulatory Commission, 5 presidents or managers from financial institutions and high-tech firms, and 2 scholars in finance-related departments, and the results as the following three perspectives (dimensions) and 20 affiliated criteria of influence are as shown in Table 1. According to existing studies and expert opinions, the factors were compiled, and this study selected the more important factors with risks, as based on triangular fuzzy numbers, with a mean of 8 and above. The important scales were kept to facilitate the design of the second phase questionnaire, including 12 government officials of the Guangdong office, the China Banking Regulatory Commission, 24 presidents or managers from financial institutions and high-tech firms, and 8 scholars in finance-related departments, with the results elaborated in Table 2. The results were subject to empirical analysis, as based on research methodology stated herein. Respondents were asked to propose the degree of direct influence that each criterion/aspect exerts on another criterion/aspect, according to a scale ranging from 0 to 4, which represent a range from ‘no influence(0)’ to ‘very high influence(4)’.

**Table 1:** The technology and financial services platform evaluation factors for the pre-test questionnaire

Perspectives (or called dimensions)		
Investment and finance platform	Credit rating platform	Credit guarantee platform
*Criteria*		
Equity and debt finance Listed finance High-tech microfinance bank High-tech corporate finance network Government support funds Trust company Trust fund Management, tracking and counseling after investment and finance	High-quality credit rating institutions Corporate and individual credit basic databases High-tech external rating databases Report from credit rating agencies Property appraisal organization	High-tech credit guarantee institution Corporate and individual credit basic databases Local government guarantee firm Re-guarantee company Private guarantee company Loan insurance Subsidy system of high-tech guarantee loss compensation

**Table 2:** Technology and financial services platform evaluation factors

Dimensions/criteria	Descriptions	References
*Investment and finance platform (A)*		
Equity and debt finance (*a*_1_)	High-tech enterprises by using equity and debt pledge to finance	Berger and Udell (1998), Brown and Petersen (2010), Bystrӧm (2008), Kaivanto and Stoneman (2007), Pretes (2002)
Listed finance (*a*_2_)	Guide and help high-tech companies by using multi-channel and higher efficiency for public finance	Berger and Udell (1998), Brown and Petersen (2010), Bystrӧm (2008)
High-tech microfinance bank (*a*_3_)	Establish a lot of bank that can provide high-tech firms microfinance	Buckley (1997)
High-tech corporate finance network (*a*_4_)	Set up a finance-related information network platform for high-tech enterprises only, including financing information, financing services, etc	Respondents
Government support funds (*a*_5_)	A special fund for high-tech company support by government, and the establishment of a sound and efficient system of support fund, such as high quality fund approval system	Cumming (2007)
Management, tracking and counseling after investment and finance (*a*_6_)	Management, tracking and counseling after investment and finance of the debt by government assisting loans	Respondents
*Credit rating platform (B)*		
High-quality credit rating institutions (*b*_1_)	Provide high quality (such as fair, independent and accurate) credit rating private agencies	Akdemir and Karsli (2012), Bannier and Hirsch (2010); Hartarska and Nadolnyak (2008)
Corporate and individual credit basic databases (*b*_2_)	Provide business and personal basic information, and loans and guarantees credit information, as well as the databases of the corporate main financial indicators	Agier and Szafarz (2013), Avery et al. (2004), Bartoli et al. (2013), Huda (2012), Oreski et al. (2012), Voordeckers and Steijvers (2006), Zhu and Zhang (2012)
High-tech external rating databases (*b*_3_)	Provide high-tech enterprises credit rating databases	Chen and Cheng (2013)
Property appraisal organization (*b*_4_)	Appraiser agencies that provide an assessment of the value of the assets, including the assessment of intellectual property rights pledge	Muller and Riedl (2002)
*Credit guarantee platform (C)*		
High-tech credit guarantee institution (*c*_1_)	Widely established the loan guarantee agencies for high-tech enterprises	Beck et al. (2005), Crowling (2010), Riding et al. (2001)
High-tech enterprises credit guarantee funds (*c*_2_)	Government set up a high-tech credit guarantee funds to help high-tech enterprises credit guarantee	Bartoli et al., (2013), Columba et al. (2010)
Local government guarantee firm (*c*_3_)	Establish high-tech credit guarantee firms of cooperation between the government and financial institutions	Honohan (2010)
Re-guarantee company (*c*_4_)	Build re-guarantee companies by government, through re-guarantee company credit enhancement, as well as the diversification of risk of guarantee	Respondents
Subsidy system of high-tech guarantee loss compensation (*c*_5_)	Guarantee institutions incurred losses for the credit guarantee of high-tech enterprises, to be compensated by the government guarantee program to a proportion of the actual loss compensation	Respondents

### Proposed MADM approach

MADM is a methodology that simultaneously weighs multiple decision-making attributes to help decision makers prioritize program attributes and make an optimal selection when given limited viable options. The methods of MADM can be applied to explore the key technology and finance services platform factors that conflict with each other. MADM procedures focus on how to select the best one among specified finite alternatives/factors (Hwang and Yoon 2012). Another advantage of MADM methods is that they have the capacity to analyze qualitative evaluation criteria. One disadvantage of MADM techniques is the high volume of calculations for finding pairwise comparison, and it is difficult to utilize them when there are a large number of criteria/factors. These techniques need arbitrary ideal levels; however they can’t match with the subjective features of the decision makers (Asgari and Abbasi 2015). This paper seek to understand whether the dimensions or criteria of technology and finance services platform interact or are independent in order to develop a complete performance measurement decision model. This study uses the DEMATEL technique to determine the effect on each dimension and criterion. Subsequently, the DANP approach, a novel combination of the DEMATEL and ANP methods based on concepts developed by Saaty (1996), is adopted to calculate the weights of the criteria. The modified VIKOR are applied to transform the performance values into gaps.

### Building an INRM by DEMATEL

The DEMATEL technique was first developed by the Geneva Research Centre (Fontela and Gabus 1976) for the purpose of showing a network relation diagram, a structural model for understanding specific societal problems (Yang and Tzeng 2011). These basic concepts were used to create a series of new hybrid MADM models and the best systematic improvement strategies for reducing the gaps for all criteria to achieve the aspiration level (Tzeng and Huang 2011; Peng and Tzeng 2013). This method is widely used to solve various types of complex problems such as mobile banking services (Lu et al. 2015) and stock performance improvement (Shen et al. 2014) and can be used to effectively understand complex structures and provide viable options for problem-solving.

The DEMATEL technique involves three steps detailed below.

*Step 1* Find the initial average matrix $\varvec{B}$. Assume that experts (respondents) G and factors n are asked to rate that the pair-wise comparisons between any two factors are denoted by, and are given, an integer score from “absolutely no influence (0)” to “very high influence (4), and showing the degree that each factor/criterion i affects each factor/criterion j. The answers by each expert fabricate a $n \times n$ non-negative matrix $\varvec{X}^{g} = \left[ {x_{ij}^{g} } \right],$ $1 \le g \le G,$ where $\varvec{X}^{1} , \ldots ,\varvec{X}^{g} , \ldots ,\varvec{X}^{G}$ are the answer matrices by the G experts, and the elements of $\varvec{X}^{g}$ are denoted by $x_{ij}^{g}$ from expert g. Thus, an $n \times n$ initial average matrix $\varvec{B}$ of all experts given is presented as in Eq. (1):

1 $$\varvec{B} = \left[ {\begin{array}{*{20}c} {b_{11} } & \ldots & {b_{1j} } & \ldots & {b_{1n} } \\ \vdots & {} & \vdots & {} & \vdots \\ {b_{i1} } & \ldots & {b_{ij} } & \ldots & {b_{in} } \\ \vdots & {} & \vdots & {} & \vdots \\ {b_{n1} } & \ldots & {b_{nj} } & \ldots & {b_{nn} } \\ \end{array} } \right]$$

The initial average scores of the *G* experts are $b_{ij} = \frac{1}{G}\sum\nolimits_{g = 1}^{G} {x_{ij}^{g} }$. The initial average matrix is called the initial direct relation matrix ***B***, and represents the degree of influence a factor exerts on another, as well as the degree of influence it receives from other factors.

*Step 2* Normalize the initial average influence-relation matrix. The normalized initial direct influence-relation matrix $\varvec{N}$ is obtained by normalizing the average matrix $\varvec{B}$. The matrix $\varvec{N}$ can be derived by Eqs. (2) and (3), whereby $\lim_{\alpha \to \infty } = [0]$.

2 $$\varvec{N} = z \cdot \varvec{B}$$

3 $$z = \hbox{min} \left\{ {\frac{1}{{\mathop {\hbox{max} }\nolimits_{i} \sum\nolimits_{j = 1}^{n} {b_{ij} } }},\frac{1}{{\mathop {\hbox{max} }\nolimits_{j} \sum\nolimits_{i = 1}^{n} {b_{ij} } }}} \right\},\quad i,j \in \{ 1,2, \ldots ,n\}$$

*Step 3* Calculate the total influence-relation matrix $\varvec{T}$. The total influence-relation matrix $\varvec{T}$ can be obtained through a summing the direct influence effects $\varvec{N}$ and indirect effects $\mathop {\lim }\limits_{\alpha \to \infty } (\varvec{N}^{2} + \ldots + \varvec{N}^{\alpha } )$. The total influence-relation matrix $\varvec{N}$ is a $n \times n$ matrix and is defined by $\varvec{T} = \left[ {t_{ij} } \right]_{n \times n} ,i,j = 1,2, \ldots ,n$ as in Eq. (4).

4 $$\begin{aligned} \varvec{T} & = \varvec{N} + \varvec{N}^{2} + \ldots + \varvec{N}^{\alpha } \\ & = \varvec{N}(\varvec{I} + \varvec{N} + \varvec{N}^{2} + \ldots + \varvec{N}^{\alpha - 1} ) \\ & = \varvec{N}(\varvec{I} + \varvec{N} + \varvec{N}^{2} + \ldots + \varvec{N}^{\alpha - 1} )(1 - \varvec{N})(1 - \varvec{N})^{ - 1} \\ & = \varvec{N}(I - \varvec{N})^{ - 1} ,\;{\text{when }}{\lim}_{\alpha \to \infty } N^{\alpha } = [0]_{n \times n} \\ \end{aligned}$$

where $\varvec{I}$ is a $n \times n$ unit matrix. The total influence-relation matrix $\varvec{T}$ of INRM can be obtained by Eq. (4). Equations (5) and (6) are used to generate each row sum (***r***) and column sum (***c***) in the matrix $\varvec{T}$, respectively.

5 $$\varvec{r} = (r_{i} )_{n \times 1} = \left[ {\sum\limits_{j = 1}^{n} {t_{ij} } } \right]_{n \times 1} = \left( {r_{1} , \ldots ,r_{i} , \ldots ,r_{n} } \right)^{{\prime }}$$

6 $$\varvec{c} = (c_{j} )_{n \times 1} = (c_{j} )^{'}_{1 \times n} = \left[ {\sum\limits_{i = 1}^{n} {t_{ij} } } \right]^{'}_{1 \times n} = (c_{1} , \ldots ,c_{j} , \ldots ,c_{n} )^{{\prime }}$$

where $r_{i}$ denotes the row sum of the *i*th row of the total influence-relation matrix $\varvec{T}$, which indicates the total effects (both direct and indirect) of factor *i* on the other factors. Similarly, $c_{j}$ denotes the column sum of the *j*th column of the total influence-relation matrix $\varvec{T}$, which indicates the total effects (both direct and indirect) of factor *j* received from the other factors. Thus, when $i = j$, $(r_{i} + c_{i} )$ provides an index of the strength of the total influences dispatched and received, that is $(r_{i} + c_{i} )$ indicates the degree of the central role that factor *i* plays in the system. In addition, $(r_{i} - c_{i} )$ provides an index of the degree of the cause of total influences. If $(r_{i} - c_{i} )$ is positive, then factor *i* gives influence upon the strength of other criteria, and if $(r_{i} - c_{i} )$ is negative, then factor *i* received influence from other criteria (Peng and Tzeng 2013; Chen and Chi 2015).

### Identifying the influential weights by DANP

The basic ANP concepts are applied in the DEMATEL method in order to confirm the influential relationship of the criteria of DANP, which shows the relative influential weights of criteria. The ANP method is considered suitable to treat complex network relationships, which is expanded from AHP (Analytic Hierarchy Process) method by Saaty (1996), Wu et al. (2013). In this paper, the DEMATEL method is adopted into the ANP method to generate the un-weighted super-matrix. Therefore, this research combined the advantages of ANP and DEMATEL to solve the problems of dependence and feedback associated with the interrelations between criteria (Chen 2015; Chen et al. 2015; Kim and Kim 2015).

The influential weights of DANP (DEMATEL–based ANP) can be described the following steps.

*Step 1* Total influence-relation matrix $\varvec{T}_{C} = [t_{ij} ]_{n \times n}$. The total influence-relation matrix $\varvec{T}_{C}$ from the total influence matrix ***T*** of DEMATEL, with different degrees of influence for the criteria is shown in Eq. (7):7where $D_{n}$ denotes the *n*th cluster; $c_{nm}$ denotes the *m*th criterion in the *n*th dimension; and $\varvec{T}_{C}^{ij}$ denotes the eigenvector of the effect of the criteria from a comparison of the *j*th dimension and the *i*th dimension.

*Step 2* Develop an un-weighted super-matrix $\varvec{Y}$. Normalize the total influence-relation matrix $\varvec{T}_{C}$ as shown in Eq. (8):8where $\varvec{T}_{C}^{\alpha }$ normalized total influence-relation matrix $\varvec{T}_{C}^{{}}$, and $\varvec{T}_{c}^{\alpha 12}$ is derived from Eqs. (9) and (10), and repeat to obtain $\varvec{T}_{c}^{\alpha nn}$.

9 $$d_{i}^{{\text{12}}} \text{ = }\sum\limits_{{j = \text{1}}}^{{m_{1} }} {t_{cij}^{{\text{12}}} },\quad i\text{ = 1, 2, } \cdots \text{, }m_{\text{1}}$$

10 $$\varvec{T}_{c}^{\alpha 12} = \begin{array}{*{20}c} {} \\ {\left[ {\begin{array}{*{20}c} {{t_{{\text{11}}}^{{\text{12}}}}/{d_{\text{1}}^{{\text{12}}} }} & \cdots & {{t_{1j}^{12} }/{d_{1}^{12} }} & \cdots & {{t_{{1m_{1} }}^{12} }/{d_{1}^{12} }} \\ \vdots & {} & \vdots & {} & \vdots \\ {{t_{{i\text{1}}}^{{\text{12}}}}/ {d_{i}^{{\text{12}}} }} & \cdots & {{t_{ij}^{12}}/{d_{i}^{12} }} & \cdots & {{{t_{{im_{1} }}^{12} }/{d_{i}^{12} }}}\\ \vdots & {} & \vdots & {} & \vdots \\ {{t_{{m_{\text{1}} \text{1}}}^{{\text{12}}}}/{d_{{m_{\text{1}} }}^{{\text{12}}} }}& \cdots & {{t_{{m_{\text{1}} j}}^{12}}/{d_{{m_{\text{1}}}}^{12} }} & \cdots & {{t_{{m_{1} m_{1} }}^{12}}/{d_{{m_{\text{1}} }}^{12}}} \\ \end{array}} \right]} \\ \end{array} = \begin{array}{*{20}c} {\left[ {\begin{array}{*{20}c} {t_{c11}^{\alpha 12} } & \cdots & {t_{c1j}^{\alpha 12} } & \cdots & {t_{{c1m_{1} }}^{\alpha 12} } \\ \vdots & {} & \vdots & {} & \vdots \\ {t_{ci1}^{\alpha 12} } & \cdots & {t_{cij}^{\alpha 12} } & \cdots & {t_{{cim_{1} }}^{\alpha 12} } \\ \vdots & {} & \vdots & {} & \vdots \\ {t_{{cm_{1} 1}}^{\alpha 12} } & \cdots & {t_{{cm_{1} j}}^{\alpha 12} } & \cdots & {t_{{cm_{1} m_{1} }}^{\alpha 12} } \\ \end{array} } \right]} \\ \end{array}$$

The un-weighted super-matrix ***Y*** can be found by transposing the normalized total matrix $\varvec{T}_{C}^{\alpha }$ by dimensions based on the concept of ANP resulting in the un-weighted super-matrix $\varvec{Y} = (\varvec{T}_{C}^{\alpha } )^{\prime}$ as shown by Eq. (11).11


*Step 3* Obtain the weighted super-matrix $\varvec{Y}^{\alpha }$. The total influence-relation matrix $\varvec{T}_{D}^{{}}$ of dimensions is derived using DEMATEL technique as given by Eq. (12):

12 $$\varvec{T}_{D}^{{}} = \left[ {\begin{array}{*{20}c} {t_{11}^{{}} } & \cdots & {t_{1j}^{{}} } & \cdots & {t_{1n}^{{}} } \\ \vdots & {} & \vdots & {} & \vdots \\ {t_{i1}^{{}} } & \cdots & {t_{ij}^{{}} } & \cdots & {t_{in}^{{}} } \\ \vdots & {} & \vdots & {} & \vdots \\ {t_{n1}^{{}} } & \cdots & {t_{nj}^{{}} } & \cdots & {t_{nn}^{{}} } \\ \end{array} } \right] .$$

The total influence-relation matrix $\varvec{T}_{D}$ divided by $d_{i} = \sum\nolimits_{j = 1}^{n} {t_{ij}^{{}} }$, $i = 1,2, \ldots ,n$, whereby I can find the normalized total influence-relation matrix $\varvec{T}_{D}^{\theta }$ of dimensions, as shown in Eq. (13).

13 $$\varvec{T}_{D}^{\theta } = \left[ {\begin{array}{*{20}c} {t_{11} /d_{1} } & \cdots & {t_{1j} /d_{1} } & \cdots & {t_{1n} /d_{1} } \\ \vdots & {} & \vdots & {} & \vdots \\ {t_{i1} /d_{i} } & \cdots & {t_{ij} /d_{i} } & \cdots & {t_{in} /d_{i} } \\ \vdots & {} & \vdots & {} & \vdots \\ {t_{n1} /d_{n} } & \cdots & {t_{nj} /d_{n} } & \cdots & {t_{nn} /d_{n} } \\ \end{array} } \right] = \left[ {\begin{array}{*{20}c} {t_{11}^{\theta D} } & \cdots & {t_{1j}^{\theta D} } & \cdots & {t_{1n}^{\theta D} } \\ \vdots & {} & \vdots & {} & \vdots \\ {t_{i1}^{\theta D} } & \cdots & {t_{ij}^{\theta D} } & \cdots & {t_{in}^{\theta D} } \\ \vdots & {} & \vdots & {} & \vdots \\ {t_{n1}^{\theta D} } & \cdots & {t_{nj}^{\theta D} } & \cdots & {t_{nn}^{\theta D} } \\ \end{array} } \right]$$

The weighted super-matrix $\varvec{Y}^{\theta }$ (normalized super-matrix) can be easily obtained by multiplying $\varvec{T}_{D}^{\theta }$ by ***Y*** as in Eq. (14):

14 $$\varvec{Y}^{\theta } = \varvec{T}_{D}^{\theta } \varvec{Y} = \;\left[ {\begin{array}{*{20}c} {t_{11}^{\theta D} \times \varvec{Y}_{11} } & \cdots & {t_{i1}^{\theta D} \times \varvec{Y}_{i1} } & \cdots & {t_{n1}^{\theta D} \times \varvec{Y}_{n1} } \\ \vdots & {} & \vdots & {} & \vdots \\ {t_{1j}^{\theta D} \times \varvec{Y}_{1j} } & \cdots & {t_{ij}^{\theta D} \times \varvec{Y}_{ij} } & \cdots & {t_{nj}^{\theta D} \times \varvec{Y}_{nj} } \\ \vdots & {} & \vdots & {} & \vdots \\ {t_{1n}^{\theta D} \times \varvec{Y}_{1n} } & \cdots & {t_{in}^{\theta D} \times \varvec{Y}_{in} } & \cdots & {t_{nn}^{\theta D} \times \varvec{Y}_{nn} } \\ \end{array} } \right]$$

*Step 4* Find the limit super-matrix $\varvec{Y}^{\theta }$. **T**he weighted super-matrix is raised to the limiting power, until it has converged and become a stable super-matrix that the global priority vector are obtained, called the DANP (DEMATEL-based ANP) influence weights $\mathop {\lim }\nolimits_{z \to \infty } (\varvec{Y}^{\theta } )^{z}$.

### Measuring the total performance by modified VIKOR

The VIKOR method, as introduced by Opricovic (1998) and developed by Opricovic and Tzeng (2007), uses the concept of compromise to solve MADM problems with conflicting criteria. It introduces the multiple criteria ranking index based on the particular measure of ‘closeness’ to the ‘ideal’ solution. The best one can be selected among the alternatives, as based on the concept of a compromise solution, when handling complex decision making problems in the MADM framework (Zeleny 1982). The modified VIKOR is applied here to derive the optimal alternative/criteria/factor; and can be divided into the following steps.

*Step 1* Derive the positive-ideal solution and negative-ideal solution replaced by the aspiration levels and the worst value. Define the best value (aspiration level) $f_{j}^{aspire}$ and the worst value $f_{j}^{worst}$ in assessment criteria, which can be obtained from traditional form to the modified form.

1. Traditional VIKOR approach
$$f_{j}^{ * } = \mathop {\hbox{max} }\limits_{l} \,\{ f_{lj} |j = 1,2, \ldots ,m\}$$ (traditional approach)
  or propose the positive-ideal solution: vector $\, \varvec{f}_{{}}^{*} = (f_{1}^{*} , \ldots ,f_{j}^{*} , \ldots ,f_{n}^{*} )$
$$f_{j}^{ - } = \mathop {\hbox{min} }\limits_{l} \,\{ f_{lj} |j = 1,2, \ldots ,m\}$$ (traditional approach)
  or propose the negative-ideal solution: vector $\, \varvec{f}_{{}}^{ - } = (f_{1}^{ - } , \ldots ,f_{j}^{ - } , \ldots ,f_{n}^{ - } )$
2. Modified VIKOR approach
  The aspiration level: $\, \varvec{f}^{aspire} = \left( {f_{1}^{aspire} , \ldots ,f_{j}^{aspire} , \ldots ,f_{n}^{aspire} } \right)$, where $f_{j}^{aspire}$ is an aspiration level;
  The worst values: $f^{worst} = (f_{1}^{worst} , \ldots f_{j}^{worst} , \ldots f_{n}^{worst} )$, where $f_{j}^{worst}$ is a worst value.

In this study, the performance scores can be obtained by using the questionnaires with a scale ranging from 0 to 10 [“very bad (0)”;“very good (10)”], so the aspiration level takes the highest score of 10 and the worst value takes value of 0. Hence, $f_{j}^{aspire} = 10$ is defined as the aspiration level and $f_{j}^{worst} = 0$ as the worst value, and can avoid choosing the best among inferior choices/options/alternatives.

*Step 2* Compute the values of the mean of group utility (average gap) $S_{l}$ and maximal regret (maximal gap) $Q_{l}$. $S_{l}$ and $Q_{l}$ can be calculated using Eqs. (16) and (17), respectively:

15 $${S_l} = \sum\limits_{j = 1}^n {{w_j}{r_{lj}}} = \sum\limits_{j = 1}^n {\left[ {{{w_j}\left( {\left| {f_j^{aspire} - {f_{lj}}} \right|} \right)}/{\left( {\left| {f_j^{aspire} - f_j^{worst}} \right|} \right)}} \right]}$$

16 $${Q_l} = \mathop {{\rm{max}}}\limits_j \left\{ {{\left( {\left| {f_j^{aspire} - {f_{lj}}} \right|} \right)}/{\left( {\left| {f_j^{aspire} - f_j^{worst}} \right|} \right)}}\left| {j = 1,2, \ldots ,n} \right. \right\}$$

where $S_{l}$ is defined as the normalized ratio of distance to the aspiration level, which implies the synthesized gap for the criteria. On the other hand, $Q_{l}$ is defined as the normalized ratio of distance to the worst value, which implies the maximal gap in *j* criteria for priority improvement. However, $\min_{l} S_{l}$ represents the maximum group utility, and $\min_{l} Q_{l}$ represents the minimum of the maximum individual regrets. Here, ${r_{lj}} = {{\left( {\left| {f_j^{aspire} - {f_{lj}}} \right|} \right)}/{\left( {\left| {f_j^{aspire} - f_j^{worst}} \right|} \right)}}$ emphasizes the normalized gap of distance to the aspiration level.

*Step 3* Compute the Index value $R_{l}$. The values are formulated as follows.

17 $$R_{l} = v(S_{l} - S^{*} )/(S^{ - } - S^{*} ) + (1 - v)(Q_{l} - Q^{*} )/(Q^{ - } - Q^{*} )$$

where $S^{*} = \mathop { \hbox{min} }\limits_{l} S_{l}$,$S^{ - } = \mathop { \hbox{max} }\limits_{l} S_{l}$, $Q^{*} = \mathop { \hbox{min} }\limits_{l} Q_{l}$, $Q^{ - } = \mathop { \hbox{min} }\limits_{l} Q_{l}$ and $0 \le \nu \le 1$, where *v* is illustrated as a weight for the strategy of maximum group utility, where 1 − *v* is the weight of the individual regret (maximal gap for priority improvement). Therefore, Eq. (17) can be rewritten as $R_{l} = vS_{l} + (1 - v)Q_{l}$ in the modified VIKOR method, when $S^{*} = S^{aspire} = 0$ and $Q^{*} = Q^{aspire} = 0$ as well as $S^{ - } = S^{worst} = 1$ and $Q^{ - } = Q^{worst} = 1$ are set.

## An empirical case: an improvement plan for Guangdong technology and financial services platform

**Fig. 2:**
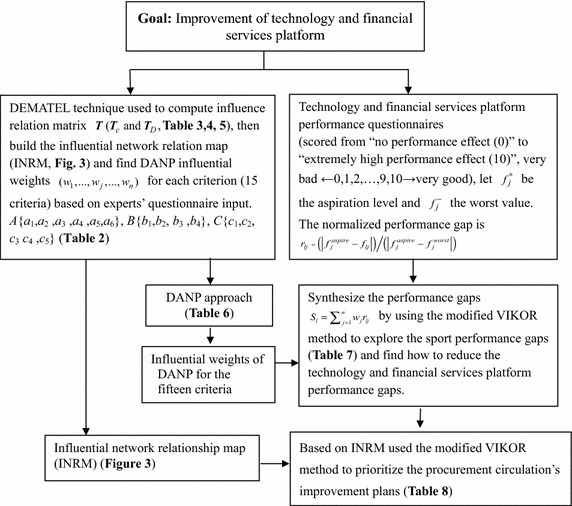
Diagram of the process for the empirical case

An empirical case study of technology and financial services performance measurements and improvements for Guangdong province is determined in this section in order to demonstrate the feasibility of the proposed model for solving real-world problems. A diagram of the empirical case study is further described in Fig. 2.

### Background and problem descriptions

The question of the insufficiencies of collateral, credit information, and economic benefits of SME loans, as well as political risks associated with SME credit, are the four main obstructions for China’s SMEs financing market (Yibin 2003). A commercial guarantee company was the first to appear in Guangdong in 1994, which established a provincial and municipal level of a government-sponsored credit guarantee agency in 1999. With support and encouragement from the central government, China’s credit guarantee schemes have been an important means for local government to ensure credit for high-tech enterprises. However, the function of the credit guarantee schemes was difficult to implement, as the intervention of local government lacked sound prudential supervision.

The main financing channel of high-tech SMEs is debt financing, such as bank credit, issuing bonds, commercial papers, etc. In order to enhance technology and finances, the Guangdong provincial government and financial institutions built a collaboration platform combining technology and finances, and established a special fund to alleviate the shortage of high-tech industries funds. The local government continued to promote technology and finance in Guangzhou, Shenzhen, Dongguan, Zhuhai, Foshan, etc., in an attempt to expand and strengthen the level of technology and financial services. Guangdong’s credit system and guarantee system were not complete, thus, information asymmetry led to low efficiency of resource allocation, and did not established a well-functioning or flexible technology and financial services platform. Financing guarantee institutions for high-tech enterprises and professional assessment organizations of technological achievements are immature, and the existence of these problems limited the provincial development of technology and finances. While Guangdong high-tech SMEs rely mainly on bank loans, the proportion of bank loans is no more than 5%, which may be attributed to inadequate technology and the financial services platform.

### Constructing an INRM by using the DEMATEL method

Based on expert interviews and questionnaire surveys, and using the approaches of Sect. “Proposed MADM approach”, the average influential matrix ***B*** is obtained by pair-wise comparisons regarding the inter-relationships between the fifteen determinants. The normalized direct relation/influence matrix ***N*** can be calculated through Eqs. (2) and (3). The total relationship matrix $\varvec{T}_{\varvec{D}}$ for the dimensions, and $\varvec{T}_{c}$ for the criteria, can be derived using Eq. (4), as shown in Tables 3 and 4. Table 4 indicates that all criteria and dimensions have an interacting relationship, where the significant confidence is 99.68% of the group consensuses of 20 respondents, which is more than 99% (see note of Table 3).

**Table 3:** Total influence-relation matrix ***T***: fourteen criteria

Criteria	*a*_1_	*a*_2_	*a*_3_	*a*_4_	*a*_5_	*a*_6_	*b*_1_	*b*_2_	*b*_3_	*b*_4_	*c*_1_	*c*_2_	*c*_3_	*c*_4_	*c*_5_
*a*_1_	0.143	0.172	0.211	0.199	0.118	0.125	0.116	0.129	0.130	0.145	0.163	0.159	0.162	0.146	0.144
*a*_2_	0.209	0.105	0.208	0.190	0.118	0.121	0.127	0.143	0.142	0.147	0.146	0.144	0.146	0.133	0.129
*a*_3_	0.207	0.151	0.156	0.205	0.119	0.141	0.138	0.156	0.148	0.150	0.165	0.163	0.160	0.147	0.145
*a*_4_	0.208	0.160	0.218	0.148	0.130	0.144	0.141	0.161	0.155	0.156	0.174	0.175	0.175	0.156	0.152
*a*_5_	0.201	0.152	0.207	0.190	0.095	0.134	0.128	0.138	0.138	0.143	0.155	0.170	0.175	0.156	0.158
*a*_6_	0.230	0.178	0.250	0.224	0.156	0.111	0.140	0.173	0.163	0.154	0.172	0.176	0.182	0.168	0.174
*b*_1_	0.298	0.232	0.304	0.279	0.194	0.205	0.139	0.217	0.213	0.214	0.246	0.240	0.240	0.223	0.211
*b*_2_	0.298	0.231	0.309	0.282	0.207	0.208	0.208	0.154	0.217	0.216	0.246	0.244	0.250	0.232	0.216
*b*_3_	0.279	0.215	0.283	0.261	0.190	0.189	0.186	0.200	0.139	0.199	0.223	0.226	0.229	0.212	0.198
*b*_4_	0.277	0.219	0.286	0.256	0.186	0.185	0.190	0.193	0.192	0.142	0.224	0.228	0.229	0.207	0.192
*c*_1_	0.224	0.155	0.249	0.219	0.138	0.147	0.145	0.158	0.152	0.157	0.130	0.180	0.190	0.175	0.160
*c*_2_	0.218	0.144	0.238	0.207	0.146	0.147	0.138	0.146	0.149	0.151	0.178	0.127	0.185	0.168	0.167
*c*_3_	0.193	0.133	0.210	0.186	0.127	0.134	0.121	0.129	0.123	0.131	0.154	0.160	0.115	0.156	0.147
*c*_4_	0.192	0.122	0.200	0.178	0.116	0.120	0.111	0.115	0.117	0.125	0.154	0.160	0.169	0.100	0.138
*c*_5_	0.195	0.132	0.206	0.181	0.137	0.122	0.108	0.115	0.119	0.119	0.150	0.162	0.166	0.160	0.098

*Average gaps* $= \frac{1}{n(n - 1)}\sum\nolimits_{i} {\sum\nolimits_{j} {\left( {\frac{{\left| {\bar{b}_{ij}^{q} - \bar{b}_{ij}^{q - 1} } \right|}}{{\bar{b}_{ij}^{q} }}} \right)} } \times 100\;\% = 0.32\;\% < 1\;\%$, i.e., significant confidence is 99.68%, where $\bar{b}_{ij}^{q - 1}$ and $\bar{b}_{ij}^{q}$ denote the average scores of the samples for *q* − 1 and *q*; and *n* denotes number of criteria, here *n* = 15 and *n* × *n* matrix. Where *q* = 44 indicates the number of experts

**Table 4:** Total influence matrix ***T***: four perspectives

Perspectives	A	B	C	Row sum ($r_{i}$)	Column sum ($c_{i}$)	$r_{i} + c_{i}$	$r_{i} - c_{i}$
A	0.174	0.143	0.143	0.460	0.660	1.120	−0.200
B	0.220	0.160	0.169	0.549	0.515	1.064	0.033
C	0.266	0.212	0.183	0.662	0.495	1.157	0.167

**Fig. 3:**
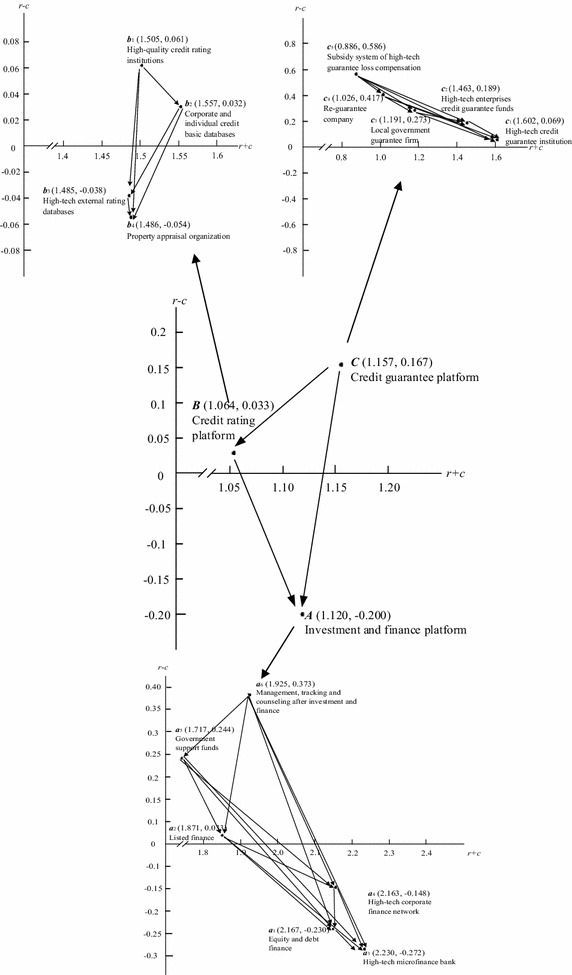
The INRM of total influence relationships for the technology and financial services platform

 Using Eqs. (5) and (6), the sums of the influence given $(r_{i} - c_{i} )$ and received $(r_{i} + c_{i} )$ by each dimension and criterion can be obtained, as shown in Table 5. Table 5 reveals that the three dimensions of investment and finance platform (*A*), credit rating platform (*B*), and credit guarantee platform (*C*) are in mutually influential relations beyond the linear relationship according to expert view. The credit guarantee platform (*C*) is first in the index of strength of influence given and received; followed by investment and finance platform (*A*), and the credit rating platform (*B*) is third. By viewing the degree of influence, the credit guarantee platform (*C*) is the dimension with the greatest influential degree ($r_{i} - c_{i} = 0.167$), the credit rating platform (*B*) is next, and investment and finance platform (*A*) is third ($r_{i} - c_{i} = - 0.2$). The credit guarantee platform dimension has obvious decisive effect on technology and financial services platform building, and it is more important than the other dimensions. The causal diagram of the total relationship, INRM, for the three dimensions and their subsystems are as presented in Fig. 3. INRM can provide information to decision-makers to easily identify improvement priorities among the complex criteria.

**Table 5:** The sum of the influences given and received on the perspectives and criteria

Perspectives/criteria	Row sum ($r_{i}$)	Column sum ($c_{i}$)	$r_{i} + c_{i}$	$r_{i} - c_{i}$
*Investment and finance platform (A)*	*0.460*	*0.660*	*1.120*	*−0.200*
Equity and debt finance (*a*_1_)	0.968	1.198	2.167	*−*0.230(5)
Listed finance (*a*_2_)	0.952	0.919	1.871	0.033(3)
High-tech microfinance bank (*a*_3_)	0.979	1.251	2.230	*−*0.272(6)
High-tech corporate finance network (*a*_4_)	1.007	1.155	2.163	*−*0.148(4)
Government support funds (*a*_5_)	0.981	0.737	1.717	0.244(2)
Management, tracking and counseling after investment and finance (*a*_6_)	1.149	0.776	1.925	0.373(1)
*Credit rating platform (B)*	*0.549*	*0.515*	*1.064*	*0.033*
High-quality credit rating institutions (*b*_1_)	0.783	0.722	1.505	0.061(1)
Corporate and individual credit basic databases (*b*_2_)	0.794	0.763	1.557	0.032(2)
High-tech external rating databases (*b*_3_)	0.723	0.761	1.485	*−*0.038(3)
Property appraisal organization (*b*_4_)	0.716	0.770	1.486	*−*0.054(4)
*Credit guarantee platform (C)*	*0.662*	*0.495*	*1.157*	*0.167*
High-tech credit guarantee institution (*c*_1_)	0.835	0.767	1.602	0.069(5)
High-tech enterprises credit guarantee funds (*c*_2_)	0.826	0.637	1.463	0.189(4)
Local government guarantee firm (*c*_3_)	0.732	0.459	1.191	0.273(3)
Re-guarantee company (*c*_4_)	0.722	0.304	1.026	0.417(2)
Subsidy system of high-tech guarantee loss compensation (*c*_5_)	0.736	0.150	0.886	0.586(1)

In Table 5, regarding influential degrees in comparison with other criteria, the ‘subsidy system of high-tech guarantee loss compensation ($c_{5}$)’ is the most important criterion ($r_{i} - c_{i} = 0.586$) for both direct and indirect influence. On other hand, the ‘high-tech microfinance bank ($a_{3}$)’ has the slightest influence on the other criteria ($r_{i} - c_{i} = - 0.272$). Furthermore, in terms of each respective dimension, ‘management, tracking, and counseling after investment and finance ($a_{6}$)’ is the most important criterion in the dimension of ‘investment and finance platform (*A*)’. At present, although a better high-tech environment exists in China, the weaker risk control ability of banks do not have a sound mechanism of management, tracking, or counseling after investment and finance, thus, there is a shortage of high-tech finance from banks. Thus, in order to improve the investment and financial platform and intensify the technology and financial services platform, it would be better to set up loan management regulations from the government to prevent non-performing loans. This would indirectly elevate bank loan wishes, while optimizing the risk and return structure and quality of high-tech finance. On the other hand, the ‘high-tech microfinance bank ($a_{3}$)’ has the least influence on the other criteria in this dimension. The ‘high-quality credit rating institutions ($b_{1}$)’ is the most important criterion in the dimension of ‘credit rating platform (*B*)’, which shows affects on the other factors. High-quality credit rating private agencies provide an objective and fair credit rating, including business, bonds, funds, individuals, etc., in order that a reliable and secure financial environment is established, which is an important factor of the development of technology and financial services. The ‘property appraisal organization ($b_{4}$)’ has the weakest influence on other factors in this dimension. The criterion of a ‘subsidy system of high-tech guarantee loss compensation ($c_{5}$)’ is the most important criterion in the dimension of ‘credit guarantee platform (*C*)’. If government could set up a compensated system for guaranteeing institutions that incur losses, the credit guarantee platform dimension would be complete. The criterion of a ‘high-tech credit guarantee institution ($c_{1}$)’ has the lowest influence on other rules in this dimension.

### Measuring the influence weights variables/criteria by using DANP

The DANP method combines ANP and DEMATEL, and conducts a survey for the case of China to facilitate the indicators of dynamic relationships. The survey results are used to create a DEMATEL-based un-weighted super-matrix, which identify the degrees of importance in the relationship, and is calculated by Eqs. (10)–(12). The weighted super-matrix is obtained by using Eqs. (13) and (14) to mirror the degrees of influence of various dimensions/criteria. The limits of the super-matrix make the weighted super-matrix raise its limiting power until it converges, and becomes a long-term stable super-matrix with the weights of the various criteria (global weight), as shown in Table 6. Based on global weight, the local weights of the assessment criteria can be derived, which helps us to comprehend the absolute weights of individual factors across all three dimensions. The primary criteria of the performance measurement of technology and financial services platform, according to the global weights, can be found.

**Table 6:** Influential weights of DANP for each criterion obtained by $\mathop { \lim }\limits_{z \to \infty } (Y^{\theta } )^{z}$

Criteria	*a*_1_	*a*_2_	*a*_3_	*a*_4_	*a*_5_	*a*_6_	*b*_1_	*b*_2_	*b*_3_	*b*_4_	*c*_1_	*c*_2_	*c*_3_	*c*_4_	*c*_5_
Weights (DANP)	0.070	0.053	0.074	0.067	0.046	0.047	0.087	0.095	0.093	0.096	0.056	0.056	0.057	0.053	0.050

### Examining performance and improvement base on modified VIKOR

The modified VIKOR method is employed to evaluate the overall technology and financial services platform performance. This study required the senior experts of financial services to assess the ameliorative effects on the respective dimensions and criteria of the technology and financial services platform in China. The score for each criterion, and the total average gap ($S_{l}$), are obtained by multiplying the respective influential weights resulted from the DANP by the gap ($r_{lj}$), as shown in Table 7. The decision-maker can then use the integrated index to find problem-solving points and improving performance for each dimension or the dimension of the criteria as a whole, and the total competitiveness gap and total performance can be obtained. To compare the other dimensions, the dimension of ‘credit rating platform (*B*)’ scored the highest (performance score = 5.467). On other hand, the dimension of ‘investment and finance platform (*A*)’ has the lowest score of the dimensions. In addition, the total performance value of the technology and financial services platform of China was 5.312. The gap from the optimum value indicates the distance of each of the evaluation criterion from the ideal/aspired level. The total gap is calculated to be 0.469, meaning that the distance from the ideal/aspired level is outpaced by 46.9%.

**Table 7:** Integrated index of technology and financial services platform dimensions and criteria

	Local weights	Global weights	Performance	Gaps ($r_{lj}$)
*Investment and finance platform (A)*	*0.357*		*5.372(2)*	*0.463(2)*
Equity and debt finance (*a*_1_)	0.197	0.070	6.625	0.338
Listed finance (*a*_2_)	0.148	0.053	7.000	0.300
High-tech microfinance bank (*a*_3_)	0.206	0.074	4.031	0.597
High-tech corporate finance network (*a*_4_)	0.188	0.067	4.563	0.544
Government support funds (*a*_5_)	0.128	0.046	5.813	0.419
Management, tracking and counseling after investment and finance (*a*_6_)	0.132	0.047	4.500	0.550
*Credit rating platform (B)*	*0.371*		*5.467(1)*	*0.453(3)*
High-quality credit rating institutions (*b*_1_)	0.236	0.087	5.000	0.500
Corporate and individual credit basic databases (*b*_2_)	0.255	0.095	5.813	0.419
High-tech external rating databases (*b*_3_)	0.252	0.093	4.625	0.538
Property appraisal organization (*b*_4_)	0.258	0.096	6.375	0.363
*Credit guarantee platform (C)*	*0.273*		*5.021(3)*	*0.498(1)*
High-tech credit guarantee institution (*c*_1_)	0.205	0.056	5.656	0.434
High-tech enterprises credit guarantee funds (*c*_2_)	0.207	0.056	4.688	0.531
Local government guarantee firm (*c*_3_)	0.211	0.057	5.250	0.475
Re-guarantee company (*c*_4_)	0.193	0.053	5.250	0.475
Subsidy system of high-tech guarantee loss compensation (*c*_5_)	0.184	0.050	4.188	0.581
Total performance	–	–	*5.312*	–
Total gap (*S*_*l*_)	–	–	–	*0.469*

### Discussion and implications

INRM was constructed based on the dimension of the DEMATEL method; this research shows that the maximum value of $r_{i} - c_{i}$ falls in the dimension of ‘credit guarantee platform (*C*)’, which implies that this dimension has the biggest influence on the other dimensions. The dimension ‘investment and finance platform (*A*)’ has the least value, denoting that this dimension is more likely than others to be affected. Since the existence of credit guarantee agencies can simplify the procedures of bank lending for high-tech enterprises, it can respond effectively to the bank’s solvency crisis, reducing the non-performing loans of banks, and therefore, stimulate banks to open up new credit business. For example, the ‘Small and Medium Enterprise Credit Guarantee Fund of Taiwan’ provides a guaranteed coverage percentage of 80–90% (Taiwan 2015), thus, banks are more willing to take risks to lend to high-tech enterprises or SMEs. Effectively operating technology and financial services, and formulating the importance ranking of each dimension and criterion, can be used as reference for improvement priorities and provide guidance for the technology and financial services platform. Based on the cause degree of $r_{i} - c_{i}$, the dimensions are ranked: *C*_*B*_*A*. In other words, the dimension of the ‘credit guarantee platform’ should have top priority for improvement.

When only the dimension of ‘credit rating platform (*B*)’ is considered, the sequence of improvement priorities can be: ($b_{1}$)_($b_{2}$)_($b_{3}$)_($b_{4}$). When only three criteria, ($b_{2}$), ($b_{3}$), ($b_{4}$), are considered in this dimension, the ranking is ($b_{2}$)_($b_{3}$)_($b_{4}$);and when only two criteria, ($b_{3}$) and ($b_{4}$), are considered, the sequenced can be ($b_{3}$)_($b_{4}$). The details of improvement priorities ranking for individual dimensions are as shown in Table 8.

**Table 8:** The technology and financial services platform implementation improvement plan

Formula	Strategy (sequence of improvement priority)
*F1* Influential network of dimensions (based on DEMATEL)	*C*_*B*_*D*
*F2* Influential network of criteria within individual dimensions	*A*: (*a*_6_)_(*a*_5_)_(*a*_2_)_(*a*_4_)_(*a*_1_)_(*a*_3_) (*a*_5_)_(*a*_2_)_(*a*_4_)_(*a*_1_)_(*a*_3_) (*a*_2_)_(*a*_4_)_(*a*_1_)_(*a*_3_) (*a*_4_)_(*a*_1_)_(*a*_3_) (*a*_1_)_(*a*_3_) *B*: (*b*_1_)_(*b*_2_)_(*b*_3_)_(*b*_4_) (*b*_2_)_(*b*_3_)_(*b*_4_) (*b*_3_)_(*b*_4_) *C*: (*c*_5_)_(*c*_4_)_(*c*_3_)_(*c*_2_)_(*c*_1_) (*c*_4_)_(*c*_3_)_(*c*_2_)_(*c*_1_) (*c*_3_)_(*c*_2_)_(*c*_1_) (*c*_2_)_(*c*_1_)

In addition, the gap between the dimensions/criteria is expressed as room for improvement, and its optimal level from the implementation performance is as shown in Table 7, the descending ranking sequence for each dimension performance is: *B*_*A*_*C*. This finding can serve as reference for decision-makers when planning for performance improvements and founding an effective technology and financial services platform. Management can understand the strengths and weaknesses of the present situation of the technology and financial services platform via this information before setting up a plan for priority improvement.

## Conclusions and remarks

This study proposed a Guangdong’s technology and financial services platform innovation strategy as an empirical case to demonstrate that the MCDM method could overcome the defects of the conventional multiple regression analysis method. First, the traditional method assumes that the factors exit an independent and hierarchic structure; however, the essence of financial markets in the real world is quite different, and a technology and financial services platform performance measurement usually relates interdependent factors. For this research, an integrated MADM method, including influential weights ANP and DEMATEL (DANP), is proposed to solve interdependence and feedback problems regarding a technology and financial services platform. Second, as aspiration levels can substitute existing alternatives to obtain the better solution fit for the current practices of the financial market; the modified VIKOR presents ideal and negative-ideal points into the aspiration level, which is used to avoid the traditional Max–Min, and choosing the best among inferior alternatives (avoid the option of ‘pick the best apple among a barrel of rotten apples’). Third, the causes of required performance improvements must be identified, and a systematic approach is used to solve dynamic problems and avoid stop-gap measures; the hybrid MADM method, as based on the INRM being derived by the DEMATEL technique, has underlying concepts of the ‘selection’ of the most preferable alternatives, which can be transferred to the ‘improvement’ of the technology and financial services platform performance in order to achieve the aspired level.

While technology and financial services in China are still in their infancy, they are also a large and complex system, and evaluation and improvement of their performance is necessary to support economic development. This study considered the present practices of financial environments, high-tech development, and experts’ opinions in order to establish an evaluation framework and indicators for a technology and financial services platform performance. This paper provides an improvement strategy for management to achieve the aspired level, which is summarized, as follows:

1. We show that the ‘credit guarantee platform (*C*)’ is the most important factor of the three dimensions, and is most likely to exert direct effect on the remaining dimensions. Consequently, the priority improvements should be: ‘credit guarantee platform (*C*)’ followed by ‘investment and finance platform (*A*)’, and finally ‘credit rating platform (*B*)’.
2. The ‘subsidy system of high-tech guarantee loss compensation ($c_{5}$)’ is the most important factor among the criteria, and is most likely to affect the other factors.
3. The dimension performance in decreasing order is: ‘credit rating platform (*B*)’, ‘investment and finance platform (*A*)’, and ‘credit guarantee platform (*C*)’.
